# Ethical-legal dilemmas of nursing practice in emergencies and disasters: a scoping review

**DOI:** 10.1590/1980-220X-REEUSP-2023-0233en

**Published:** 2024-04-15

**Authors:** Alex Coelho da Silva Duarte, Sandra Conceição Ribeiro Chicharo, Thiago Augusto Soares Monteiro da Silva, Alexandre Barbosa de Oliveira

**Affiliations:** 1 Universidade Federal do Rio de Janeiro, Escola de Enfermagem Anna Nery, Programa de Pós-Graduação em Enfermagem. Rio de Janeiro, RJ, Brazil.; 2 Universidade Federal do Rio de Janeiro, Escola de Enfermagem Anna Nery, Departamento de Enfermagem Médico Cirúrgica. Rio de Janeiro, RJ, Brazil.

**Keywords:** Disasters, Ethics, Professional Competence, Science of Disaster, Emergency Nursing, Desastres, Ética, Competencia Profesional, Ciencia del Desastre, Enfermería de Urgencia, Desastres, Ética, Competência Profissional, Ciência do Desastre, Enfermagem em Emergência

## Abstract

**Objective:**

To map the ethical-legal dilemmas related to nursing practice in emergency and disaster situations.

**Method:**

A scoping review developed in accordance with the JBI method, whose information sources were accessed in databases, in addition to gray literature. The selection was made by reading the titles, abstracts and descriptors, observing eligibility criteria, including two reviewers and a third in case of discrepancies. After reading, data extraction and content analysis of 17 selected studies were carried out.

**Results:**

Thirteen ethical-legal dilemmas were mapped related to professional/functional duty towards the family, lack of personal protective equipment and unsafe conditions at work, preparation and availability for action, skills, limits imposed by victims’ religion, obligation to provide care.

**Conclusion:**

Professionals, researchers and representatives of the category need to resolve demands that involve acting in emergencies and disasters, (re)cognizing the ethical-legal dilemma, and seeking (re)legal frameworks and observance of the fundamental/ethical principles that govern the profession, in the sense to support decision-making and the development of legally safe practices.

## RESUMO

**Objetivo:**

Mapear os dilemas ético-legais relacionados à prática de Enfermagem em situações de emergências e desastres.

**Método:**

Revisão de escopo, desenvolvida em conformidade com o método JBI, cujas fontes de informação foram acessadas em bases de dados, além da literatura cinzenta. A seleção se deu pela leitura dos títulos, resumos e descritores, observando critérios de elegibilidade, contando com dois revisores e um terceiro em caso de divergências. Após a leitura, procedeu-se à extração dos dados e análise de conteúdo de 17 publicações selecionadas.

**Resultados:**

Mapearam-se 13 dilemas ético-legais relacionados ao dever profissional/funcional diante da família, falta de equipamento de proteção individual e condição insegura no trabalho, preparação e disponibilidade para atuação, competências, limites impostos pela religião das vítimas, obrigatoriedade em prestar cuidado.

**Conclusão:**

Profissionais, pesquisadores e representantes da categoria precisam resolver demandas que envolvem a atuação em emergências e desastres, (re)conhecendo os dilemas ético-legais e buscando (re)enquadramentos legais e observância dos princípios fundamentais/éticos que regem a profissão, no sentido de amparar a tomada de decisão e o desenvolvimento de práticas seguras juridicamente.

## RESUMEN

**Objetivo:**

Mapear los dilemas ético-legales relacionados con la práctica de enfermería en situaciones de emergencia y desastre.

**Método:**

Revisión de alcance, desarrollada de acuerdo con el método JBI, a cuyas fuentes de información se accedió en bases de datos, además de literatura gris. La selección se realizó mediante lectura de títulos, resúmenes y descriptores, observando criterios de elegibilidad, contando con dos revisores y un tercero en caso de discrepancias. Luego de la lectura, se realizó la extracción de datos y análisis de contenido de 17 publicaciones seleccionadas.

**Resultados:**

Fueron mapeados 13 dilemas ético-legales relacionados con el deber profesional/funcional hacia la familia, falta de equipos de protección personal y condiciones inseguras en el trabajo, preparación y disponibilidad para la acción, habilidades, límites impuestos por la religión de las víctimas, obligación de brindar cuidados.

**Conclusión:**

Profesionales, investigadores y representantes de la categoría necesitan resolver demandas que involucran actuar en emergencias y desastres, (re)conociendo los dilemas ético-legales y buscando el (re)encuadre legal y el cumplimiento de los principios fundamentales/éticos que rigen la profesión, con el fin de apoyar la toma de decisiones y el desarrollo de prácticas jurídicamente seguras.

## INTRODUCTION

Nursing legislation involving emergencies and disasters is a complex and controversial topic. Discussing it is a fundamental task, but also a laborious one to manage, considering that disaster phenomena, whether of natural or technological origin, generally exceed the local response capacity, sometimes requiring external and even international help, when it is commonly imposed the ready employment of nursing professionals from different origins, cultures and training processes, whose practices are guided by their own and even divergent legislation^(1)^.

For the preliminary approach to this problem, we started from the definition of an ethical-legal dilemma as being one that puts nursing professionals faced with a choice, usually to do everything possible to save a life, in contrast to the doubt of “can I”, created by the absence of its own legislation, which does not have specific standards aimed at these professionals in emergencies and disasters, making it necessary to use analogies with other legal standards^(2)^.

It should be added that the reactive nature of laws in Brazil tends to impact the quality and effectiveness of emergency and disaster risk management processes, limiting or hindering institutions’ and sectors’ action, which encounter difficulties due to the lack of a legal basis. On the other hand, precarious governance at its different levels (municipal, state and federal) reinforces circumstances of vulnerability in communities, especially the most impoverished. In general, what is commonly observed is that the problem first occurs and then the Legislative Power manifests itself, regulating certain conducts with the aim of avoiding “new risks” when, in fact, the ideal would be to act in a preventive manner under the form of prospective risk management, as highlighted by the Sendai Framework for Disaster Risk Reduction 2015-2030, of which Brazil is a signatory country^(3,4)^.

Such consideration is justified, for instance, by the enactment of Law 10,308/2001, which provided for site selection, construction, licensing, operation, inspection, costs, compensation, civil liability and guarantees relating to radioactive waste deposits. We also need to mention the inclusion of Article 21, item XXIII, paragraphs “b”, “c” and “d”, and Article 177, item V, of the Constitution of the Federative Republic of Brazil of 1988, both after the radioactive accident with cesium-137 in the municipality of Goiânia, which occurred on 09/13/1987, which affected hundreds of citizens^(5,6)^.

Another example is Law 12,608/2012, which established the Brazilian National Civil Protection and Defense Policy, which provided for the Brazilian National Civil Protection and Defense System and the Brazilian National Civil Protection and Defense Council, authorizing the creation of an information and disaster monitoring. This device was created only after the floods and landslides that occurred in the mountainous region of the state of Rio de Janeiro in January 2011, a mega socio-environmental disaster that caused more than 1,000 deaths and left thousands of citizens homeless and displaced^(7)^.

Another example that endorses reactive management is Law 13,425/2017, which established general guidelines on measures to prevent and combat fires and disasters in establishments, buildings and public meeting areas, a legal instrument that was structured only after the dramatic fire occurred at *Boate* Kiss, in the municipality of Santa Maria on 01/27/2013, which left 242 dead and 636 injured, whose perpetrators have not yet been legally punished^(8)^.

In the field of nursing, the body responsible for supervising professional practice in the country, the Federal Nursing Council (COFEn – *Conselho Federal de Enfermagem*), does not establish clear standards on the subject. In its legislation database, there were 1,841 standards until February 2023, such as decisions, decrees, orders, laws, technical notes, service orders, opinions, among others. When refining the search using the terms “emergencies”, “disaster(s)” and “catastrophe(s)”, no documents were recovered. When redoing the search with the word “emergency”, only five documents were recovered, two opinions and three resolutions, which did not directly deal with legal powers to act in emergencies and disasters^(9)^.

The challenges are amplified as the various professional bodies of other health professionals issue standards regulating procedures, making them exclusive to their class on an individual basis, without prior interactions with other professional classes in the health sector. Interestingly, in the text of their rules, they exclude cases of emergencies, but do not define them precisely, leaving gaps for divergent legal interpretations.

Globally, the International Council of Nurses has been (re)defining specific competencies for nurses in disasters based on the logic of the disaster cycle, considering practices before, during and after these events, while demonstrating its commitment to bringing together professionals and researchers from different countries to amplify the debate on the need to internationally standardize the conduct adopted by nurses in disasters. However, no solutions have yet been identified to face the ethical-legal dilemmas observed in such situations^(10–12)^.

In effect, these aspects tend to cause legal uncertainty and, consequently, encourage the development of ethical-legal dilemmas when nurses work in emergencies and disasters, which can culminate in a profound impact on the decision on the conduct to be adopted and, in ultimately, in the health and safety of those affected, precisely at times when the commitment of these professionals is most needed.

This research aimed to map the ethical-legal dilemmas related to nursing practice in emergency and disaster situations.

## METHOD

This is a scoping review, which follows JBI^(13)^ method and Preferred Reporting Items for Systematic reviews and Meta-Analyses extension for Scoping Reviews (PRISMA-ScR) guidelines^(14)^. The review protocol was registered in the Open Science Framework (https://osf.io/zgpfw/) with DOI 10.17605/OSF. I/O/ZGPFW^(15)^. This scoping review aims to answer the following question: what are the ethical-legal dilemmas related to nursing practice in emergency and disaster situations?

It is noteworthy that scoping reviews map the existing evidence on a given subject, without analyzing the methodological quality of included studies, as they do not aim to find the best evidence, but to define how it was produced and in what contexts^(14,16)^. To this end, a methodological strategy consisting of six steps was followed: 1) research question identification; 2) relevant study identification; 3) study selection; 4) data extraction; 5) separation, synthesis and reporting of results; and 6) presentation of results^(17)^.

Considering the exposed problem, a preliminary search was carried out in November and December 2021 in the MEDLINE (via PubMed), PROSPERO, Cochrane Database of Systematic Reviews and JBI Evidence Synthesis databases, in which no published or ongoing reviews were identified on the topic, which supported the need for this study with the premise of mapping concepts systematically.

The mapping, (re)knowledge and scientific approach to these dilemmas may shed light on possible legal solutions and support in the (re)definition of future regulations that bring more legal security to nursing professionals, when acting in emergencies and disasters, possibly ensuring less limits on emergency care provided to individuals, families and communities affected by such events.

### Inclusion Criteria

The following guiding question was defined for the scoping review: what are the ethical-legal dilemmas related to nursing practice in emergency and disaster situations? Study search and selection for the scoping review were based on the acronym PCC. Thus, “P” (Population) refers to nursing professionals; “C” (Concept), to ethical-legal dilemmas related to nursing practice; and “C” (Context), to emergencies and disasters, whether of any origin, such as natural (floods, floods, landslides, droughts, epidemics, pandemics, infestations/pests, earthquakes, tsunamis), technological (urban fires, rupture of tailings dams, building collapses, water contamination, passenger transport accidents, accidents with explosive, chemical, biological, radiological and nuclear materials), or social (terrorist attacks, forced migrations, violence and chaos urban, wars, civil conflicts, ethnic and religious intolerance, extreme poverty, lack of assistance).

### Search Strategy

After defining the PCC acronym elements, the search strategy began by identifying terms by language in DeCS, MeSH and Emtree vocabularies. From the preliminary search, the text words contained in the titles and abstracts of relevant articles and the subject terms used to describe the articles were also considered to develop the full search strategy (Chart 1). The search strategy, including all identified keywords and index terms, was applied and adapted for each database and/or information source included: Regional Virtual Health Library Portal: “LILACS” *OR* “BDENF” *OR* “IBECS” *OR* “WHOLIS” *OR* “*campus virtual sp Brasil*” *OR* “*colecionaSUS*” *OR* “CUMED” *OR* “LIPECS” *OR* “RHS” *OR* “BINACIS” *OR* “SES-SP”, SciELO, PubMed/NLM and CAPES Portal of Journals: CINAHL, *SocINDEX*, Academic Source/EBSCO, *CAB Direct*, WoS/Clarivate Analytics, APA PsycInfo, and EMBASE and ScopUSA/ELSEVIER, in addition to the gray literature of these resources. Language and search period were not defined. Searches in databases were created on November 13, 2023 with the help of a librarian, according to the sets of terms in Chart 1.

**Chart 1:** Search strategy in the MEDLINE/PubMed database – Rio de Janeiro, RJ, Brazil, 2023.

Search	Terms	Results
#1	Search: “Nurses”[mh] OR Nurse*[tiab] OR “Nursing”[mh] OR Nursing*[tiab] OR “Ethics, Nursing”[mh] OR “Nursing Ethic”[tiab] OR “Nursing Ethics”[tiab] OR “Legislation, Nursing”[mh] OR “Nursing Legislation”[tiab] OR “Nursing Legislations”[tiab]	686,833
#2	Search: “Ethics”[mh] OR Ethic*[tiab] OR “Situational Ethics”[tiab] OR Moral Polic*[tiab] OR Natural Law*[tiab] OR Egoism[tiab] OR Metaethic*[tiab] OR “Ethics, Professional”[mh] OR Professional Ethic*[tiab] OR “Liability, Legal”[mh] OR Legal Liabilit*[tiab] OR Tort*[tiab] OR Personal Liabilit*[tiab] OR Professional Liabilit*[tiab] OR Institutional Liabilit*[tiab] OR Medical Liabilit*[tiab] OR “Malpractice”[mh] OR Negligence*[tiab] OR “Moral Obligations”[mh] OR “Moral Obligation”[tiab] OR Moral Dut*[tiab] OR “ethical conflicts”[tiab] OR legality[tiab] OR ethical dilemma*[tiab] OR “ethical quandaries”[tiab] OR “moral dilemma”[tiab] OR “moral distress”[tiab] OR “moral doubt”[tiab] OR “moral philosophy”[tiab] OR “wedge argument”[tiab] OR “physician impairment”[tiab] OR “professional impairment”[tiab] OR “Jurisprudence”[mh] OR jurisprudence[tiab] OR Constitutional Law*[tiab] OR Court Decision*[tiab] OR Law[tiab] OR Laws[tiab] OR Legal Aspect*[tiab] OR Legal Obligation*[tiab] OR “Legal Status”[tiab] OR Litigation*[tiab] OR “Medical Jurisprudence”[tiab] OR State Interest*[tiab] OR “Disaster Legislation”[tiab] OR “Legal Process”[tiab] OR “Civil Rights”[mh] OR “Civil Right”[tiab] OR Minority Right*[tiab] OR Legal Right*[tiab] OR Voting Right*[tiab] OR “Due Process”[tiab] OR “Equal Protection”[tiab] OR “legal context”[tiab] OR “ethical dilemmas”[tiab] OR “ethical dilemma”[tiab] OR “Ethical decision”[tiab] OR “legal challenges”[tiab] OR “Moral experience”[tiab] OR “ethical challenges”[tiab] OR “ethical-legal dilemma”[tiab]	602,570
#3	Search: “Disasters”[mh] OR Emergencies[mh] OR Disaster*[tiab] OR Emergencies[mh]OR catastrophe*[tiab] OR catastrophic accident*[tiab] OR Calamity[tiab] OR Tragedies[tiab]OR Sinister[tiab] OR Urgence*[tiab] OR Urgenc*[tiab] OR “Mass Casualty Incidents”[mh] OR “Mass Casualty Incident”[tiab] OR “Mass Casualties”[tiab] OR “Mass Casualty”[tiab] OR “Chernobyl Nuclear Accident”[mh] OR Fukushima Nuclear Accident*[tiab] OR Chernobyl Nuclear Accident*[tiab] OR Chornobyl Nuclear Disaster*[tiab] OR Fukushima Nuclear Disaster*[tiab]	148,406
#4	Search: #1 AND #2 AND #3	376

### Font Selection

The search results were imported into the Endnote v.X9 reference manager (Clarivate Analytics, PA, USA) in order to identify and suppress duplications. Therefore, 3,317 studies were excluded. Subsequently, the remaining 5,977 were exported to the Qatar Computing Research Institute (QCRI) Rayyan application.

A further 68 repeated articles were identified and, with the analysis carried out by the reviewers, five more were excluded for the same reason. For analysis and selection by title and abstract, which was carried out by two independent reviewers maintaining the blinding process, 5,904 studies were considered.

When analyzing the titles and abstracts, it was identified that the selected studies brought some conceptual confusion between ethical-legal dilemmas and crimes of imprudence, malpractice and negligence, which gave rise to consensus meetings between the reviewers.

After these consensus meetings, an absolute number of 5,606 articles were excluded from title and abstract analysis, as they did not meet the inclusion criteria, such as all sources of information available in the databases that address the topic of study, without idiomatic and temporal cuts. With regard to the exclusion criteria, sources that were not available in full text were disregarded, which could sometimes be overcome with attempts to communicate with their authors for access. No divergences were found between the reviewers that would require the action of a third reviewer.

The articles were organized in an instrument in Microsoft Excel^®^, which was adapted in accordance with JBI^(13)^ methodology, based on publication characterization data (year, source of information, authors, title, design, method, language, country, descriptors/keywords).

### Data Analysis, Extraction and Presentation

After critical and detailed reading of selected studies, data was extracted into a Microsoft Excel^®^ file, which was related to the description of the ethical-legal dilemmas that were mapped. The dilemmas were presented in the form of a chart.

It is noteworthy that, as it was a scoping review, which used studies and, it was not necessary to be assessed by a Research Ethics Committee.

## RESULTS

**Figure 1.**
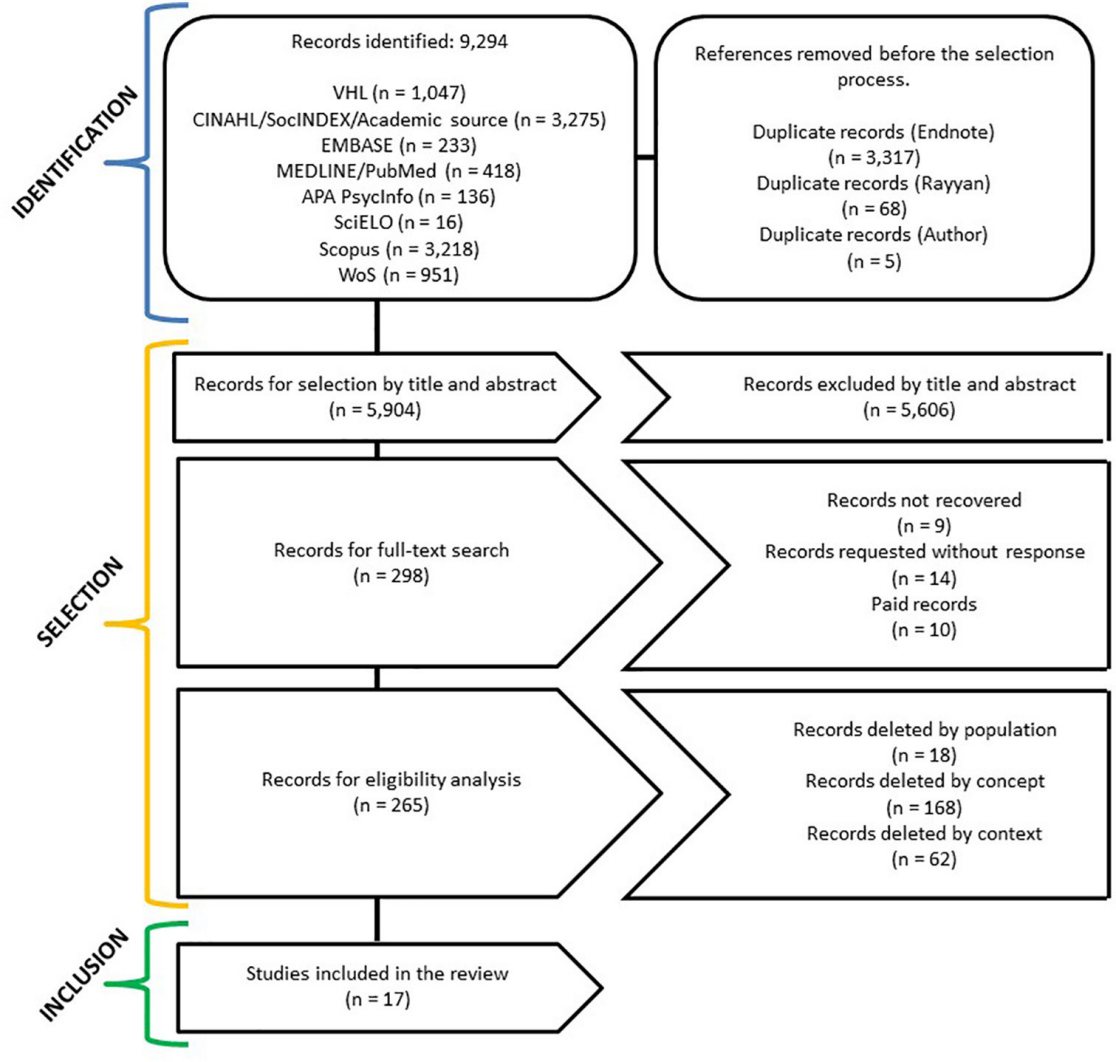
Study search and selection flowchart – Rio de Janeiro, RJ, Brazil, 2022.

The text search and selection process resulted in the inclusion of 17 studies as shown in Figure 1.

From the analysis of the 17 included studies (Chart 2), it was noted that all were produced by different authors, and that 15 (88.24%) had their text available in English, one (5.88%) in Portuguese and one (5.88%) in Italian. The largest number of studies occurred in 2008, totaling four (23.53%) studies, followed by 2017 with two (11.76%) studies, while the other years recorded one (5.88%) study each.

**Chart 2:** Selected sources of information – Rio de Janeiro, RJ, Brazil, 2023.

№	Source/year	Authors	Title	Design/method	Country/language	Keywords
01	Academic Emergency Medicine/2002^(18)^	A.W. Beckman, B.K. Sloan, G.P. Moore, W.H. Cordell, E.J. Brizendine, E.T. Boie, et al.	Should parents be present during emergency department procedures on children, and who should make that decision? A survey of emergency physician and nurse attitudes	Article/field research	USA/English	Emergency Medicine; Ethics; Pain; Parental Presence; Pediatrics; Procedures
02	Italian Heart Journal Supplement/2005^(19)^	M. Giammaria, W. Frittelli, R. Belli, A. Chinaglia, B. De Michelis, S. Ierna, et al.	Does reluctance to perform mouth-to-mouth ventilation exist among emergency healthcare providers as first responders?	Article/field research	Italy/Italian	Cardiopulmonary Resuscitation; First Responder
03	Nursing/2006^(20)^	S.A. Salladay	Putting your life on the line	Note to editor/reflection	USA/English	Not applicable
04	*Biblioteca Digital da USP*/2008^(21)^	A.L.T. Dolor	Atendimento pré-hospitalar: histórico do papel do enfermeiro e os desafios ético-legais	Dissertation/document analysis	Brazilian/Portuguese	*Emergência; Enfermagem; Ético-Legais; História da Enfermagem; Pré-Hospitalar*
05	Australian Nursing Journal/2008^(22)^	M.J. Johnstone	Emergency situations and refusals to care	Article/editorial	Australia/English	Not applicable
06	The Journal of the New York State Nurses’ Association/2008^(23)^	A.L. Pacsi	Case study: an ethical dilemma involving a dying patient	Article/case study	USA/English	Not applicable
07	Disaster Medicine and Public Health Preparedness/2008^(24)^	J. Tabery, C.M. Macckett	Ethics of triage in the event of an influenza pandemic	Article/essay	USA/English	Direct Multiplier Effect; Ethics; Influenza Pandemic; Public Health; Triage; Utility
08	International Disaster Nursing/2010^(25)^	L.D. Toiviainen, E. Daily	Disaster ethics	Book chapter/descriptive	Australia/English	Not applicable
09	Journal of Emergency Management/2013^(26)^	O.W. Fung, A.Y. Loke	Nurses’ willingness and readiness to report for duty in a disaster	Article/cross-sectional description	China/English	Disaster Nursing Management; Hong Kong Nurses; Readiness to Work; Willingness to Report for Duty; Workforce Management
10	Journal of Nursing Management/2014^(27)^	M. Ben Natan, S. Nigel, I. Yevdayev, M. Qadan, M. Dudkiewicz	Nurse willingness to report for work in the event of an earthquake in Israel	Article/self-administered questionnaire	Israel/English	Earthquake; Israel; Nurse; Willingness
11	Nursing Ethics/2015^(28)^	F. Aliakbari, K. Hammad, M. Bahrami, F. Aein	Ethical and legal challenges associated with disaster nursing	Article/descriptive study	Iran/English	Competencies; Iran; Disaster; Ethics; Law; Nurse; Professional Responsibility
12	MedSurg Matters Newsletter/2017^(29)^	C.J. Cassidy	A Nurse’s Ethical Obligation During a Pandemic	Article/descriptive study	USA/English	Ethics, Nursing; Disease; Outbreaks; Nurse Attitudes
13	Nursing Standard/2017^(30)^	I. Dowie	Legal, ethical and professional aspects of duty of care for nurses	Article/descriptive study	Wales/English	Accountability; Duty of Care; Ethical Issues; Legal Issues; Negligence; Professional Issues; Standards of Care
14	Critical Care Medicine/2018^(31)^	T.J. Papadimos, E.G. Marcolini, M. Hadian, G.E. Hardart, N. Ward, M.M. Levy, et al.	Ethics of outbreaks position statement. part 1: Therapies, treatment limitations, and duty to treat	Article/literature review	USA/English	Disease Outbreaks; Ethics; Experimental Therapies; Medical Research; Moral Duty; Public Health
15	Nursing Ethics/2020^(32)^	C. McNeill, D. Alfred, T. Nash, J. Chilton, M.S. Swanson	Characterization of nurses’ duty to care and willingness to report	Article/ cross-sectional descriptive study	USA/English	Survey; Disaster Planning; Duty to Care; Nursing Ethics; Willingness to Report
16	Nursing Ethics/2022^(33)^	X.X. Liu, Y. Chen, Y. Chen, C. Wu, Q. Xu, H. Zhu, P. Waidley, E. Waidley	Ethical dilemmas faced by frontline support nurses fighting COVID-19	Article/phenomenological study	China/English	COVID-19; Ethical Dilemmas; Frontline Nurses; Qualitative Research; Teamwork
17	Prehospital Disaster and Medicine/2022^(34)^	R. Fairley, T. Emanuel, B. Goettl	Staff Augmentation during Disaster Response	Article/description	USA/English	Ethics; Medicolegal; Surge Staffing

Regarding the country of origin, eight (47.06%) studies were identified from the United States of America (USA), two (11.76%) from Australia, two (11.76%) from China, one (5.88%) from Brazil, one (5.88%) from Iran, one (5.88%) from Israel, one (5.88%) from Italy and one (5.88%) from the country of Welsh. As for the source, the journals that published the most on the subject were Nursing Ethics, with three (21.43%) studies, and Journal of Nursing Management, with two (11.76%).

In relation to typology, scientific articles predominated, which was observed in 14 (82.35%) studies, one (5.88%) master’s dissertation, one (5.88%) book chapter and one (5.88%) note to editor. Regarding the approach, they are all qualitative. As for the method used, descriptive studies predominated, seven (41.18%), and field research, with two (11.76%) studies. As for reflection, document analysis, editorial, case study, essay, self-administered questionnaire and literature review, all had one (5.88%) study each.

Regarding the types of disasters, the selected articles addressed Hurricane Katrina, the earthquake in Israel, car accidents and public health emergencies such as outbreaks of infectious diseases, namely the influenza pandemic and the COVID-19 pandemic, in addition to the articles that dealt with the topic without a specific focus, allowing its discussion for natural or technological disasters.

By reading the full articles, the ethical-legal dilemmas that were addressed in each study were identified and described. From this process, it was observed that some dilemmas were repeated and, for this reason, the thematic grouping of these dilemmas was carried out. Thus, as can be seen from Chart 3, data extraction resulted in the mapping of 13 ethical-legal dilemmas.

**Chart 3:** Selected sources and mapped ethical-legal dilemmas – Rio de Janeiro, RJ, Brazil, 2023.

No. dilemma	Document number	Title of reference source and year of publication	Ethical-legal dilemma
01	05	Emergency situations and refusals to care (2008)^(22)^	Professional/functional duty in the face of family demands: obligation to report to work in cases of emergency/disaster, what is the priority? Profession or family?
	09	Nurses’ willingness and readiness to report for duty in a disaster (2013)^(26)^	
	12	Nurse’s Ethical Obligation During a Pandemic (2017)^(29)^	
	14	Ethics of Outbreaks Position Statement. Part 1: Therapies, Treatment Limitations, and Duty to Treat. Critical care medicine (2018)^(31)^	
	15	Characterization of nurses’ duty to care and willingness to report (2020)^(32)^	
02	10	Nurse willingness to report for work in the event of an earthquake in Israel (2014)^(27)^	Availability to work in disasters: does concern about family members regarding the lack of medication during disasters interfere with professionals’ ability to be prepared?
03	03	Putting your life on the line (2006)^(20)^	Personal safety in disasters: to what extent is the balance between professionals’ personal safety and the care provided?
04	02	Does reluctance to perform mouth-to-mouth ventilation exist among emergency healthcare providers as first responders? (2005)^(19)^	Lack of personal protective equipment and unsafe conditions at work: can professionals refuse to work due to lack of personal protective equipment (e.g., mouth-to-mouth resuscitation)? Would this be a justified refusal? Is there a risk of reducing the number of providers?
	05	Emergency situations and refusals to care (2008)^(22)^	
05	07	Ethics of triage in the event of an influenza pandemic (2008)^(24)^	Technical preparation to act in disasters: can/should professionals act even without specific preparation?
	08	Disaster Ethics (2010)^(25)^	
	10	Nurse willingness to report for work in the event of an earthquake in Israel (2014)^(27)^	
06	06	Case study: an ethical dilemma involving a dying patient (2008)^(23)^	Religious status of victims: “Jehovah’s Witness”. Can refusal due to religious belief override life-sustaining intervention? (e.g., victim with internal bleeding requiring surgery and blood transfusion)
07	01	Should parents be present during emergency department procedures on children, and who should make that decision? A survey of emergency physician and nurse attitudes (2002)^(18)^	Minor victims in emergencies/disasters: whether or not to allow parents to be present during procedures or transportation? Who makes this decision?
08	16	Ethical dilemmas faced by frontline support nurses fighting COVID-19 (2022)^(33)^	The issue of skills: can professionals work without the appropriate specialization? (e.g., case of the COVID-19 pandemic in which professionals from other sectors, such as emergency and other clinics, were transferred to Intensive Care Units)
	17	Staff Augmentation during Disaster Response (2022)^(34)^	
09	04	*Atendimento pré-hospitalar: histórico do papel do enfermeiro e os desafios ético-legais* (2008)^(21)^	Challenges in pre-hospital care (definitive airway in situations where supraglottic devices are not available): what are the limits between clinical necessity and legality in cases of emergencies/disasters?
10			Challenges in pre-hospital care (interruption of cardiopulmonary resuscitation): who makes the decision? When to decide to interrupt?
11	13	Legal, ethical and professional aspects of duty of care for nurses (2017)^(30)^	Regarding duty of care: when witnessing an emergency/disaster situation, is there an obligation on the professional to provide care to the victims?
12			Regarding duty of care: in an emergency/disaster situation, such as a terrorist attack or a significant fire, is it reasonable for an off-duty nurse to put herself in personal danger to protect the lives of others? Would it be fair or merely reasonable in such circumstances to impose a duty of care on nurses?
13	11	Ethical and legal challenges associated with disaster nursing (2015)^(28)^	Problems related to limits of professional practice: at the scene of the disaster, a professional realizes that they need to carry out an invasive procedure that they believe is not authorized from the point of view of professional standards. Knowing that there is a medical professional on site, even in a chaotic disaster situation, can/should the professional carry out the procedure? Does the presence of a medical professional affect the decision?

## DISCUSSION

The analysis of identified sources demonstrated weaknesses in conceptual uses in typification of ethical-legal dilemmas in relation to dissatisfactions with the profession and intentional crimes, i.e., those committed by acts of malpractice, imprudence and negligence, which constitutes relevant data that can have implications not only for the scientific field, as such concepts are not adequately addressed, but also for professionals and legal fields, which consume academic subjects.

Furthermore, when specificities involving nursing practices, peculiarities regarding the nature, the technical division of labor and specific competencies of this profession are recognized, on a global scale, the problem tends to be magnified, since the different formats and quality of training and employability processes have potential implications for interventions in emergencies and disasters, understanding that such phenomena involve, in many cases, the need for international aid, when the country’s response capacity achieved is overcome. Thinking in this sense implies understanding the need to standardize skills to act in such events as well as to have a framework and legal framework designed and structured in cooperation networks to provide support to the professionals involved on a national and international scale.

As an illustration, in the specific case of Brazil, there are no specific standards regarding nurses’ practice in emergencies and disasters in the legal framework that could well guide these professionals’ actions, leaving class representation bodies free to define standards not specified by law, which can result in conflicts that end up being judicialized, since these standards are not always guided by broad and qualified discussion by experts.

These situations tend to lead nursing professionals to face ethical-legal dilemmas, which refer to the psychological impact of having to act differently from what feels morally, ethically or professionally appropriate^(35)^. In other words, nursing professionals may be in doubt about the legality of a certain action or procedure that they feel safe to perform, and may end up not carrying it out, even though it could make the difference between life and the death of people, considering that the premise of care in emergencies/disasters is to save/maintain as many lives as possible^(35)^.

The first dilemma highlighted in Chart 3 is related to Hurricane Katrina, which occurred in the USA in 2005, in which nursing professionals were in doubt between their own need to take care of their family and their duty to report to work before of facing that disaster. A situation was portrayed in which the family would remain exposed in a risk area, vulnerable to disaster, while professionals would have to report for work. This dilemma is also something commonly observed in natural threats involving floods and landslides caused by rainfall, in which professionals living in risk areas may need to leave their homes, leaving their families in a vulnerable situation^(22,26,29,31,32)^.

The second dilemma refers to professionals’ concern with the lack of medications when facing a public health emergency or disaster and whether this may interfere with their readiness for work^(27)^. These two dilemmas reinforce the importance of identifying the factors that lead to greater team engagement in whether or not to present themselves in emergency and disaster situations, linked to management strategies to strengthen nursing work as a multidisciplinary team. It was evident that professional experience and level of knowledge increase the willingness to face these dilemmas.

Some mapped dilemmas are linked to the shortage of personal protective equipment (PPE) that imposes on professionals the doubt between providing care to victims and their own safety. Keeping the due proportions, such dilemmas involve a situation of overlapping risks, such as contamination of professionals, patients and the environment, as well as those related to the emergency/disaster scene and circumstance itself ^(19,20,22)^.

This problem was explicitly experienced during the response to the COVID-19 pandemic, when countries were faced with an insufficient supply of PPE to allow for expected exchanges during assistance, which prolonged the teams’ stay in direct contact with patients and prevented breaks to meet professionals’ basic human needs, such as hydration, food, hygiene, vesical-intestinal elimination. This fact imposed the use of disposable diapers in an attempt to reduce service interruptions and PPE changes. It was also evident that some of these devices had undue weight or even questionable standardization regarding exchange and dispensing in terms of hours of use and exchange recommendations^(36)^.

One of the most delicate and intriguing dilemmas refers to the situation of patients of the Jehovah’s Witnesses religion, positioning professionals between issues involving religious belief and survival. The selected article goes beyond blood transfusion, addressing the case of a pregnant woman, victim of a traffic accident, who needs surgery and transfusion, and who refuses both procedures, i.e., in addition to the trauma victim’s life (mother), the fetus’ life is also at risk^(23)^. It is important to understand that it is necessary to explain to patients, even if they are experiencing an emergency/disaster situation, their diagnosis, prognosis, therapeutic options and possible results and complications through the decision and conduct taken. Such action involves ethical and humanistic aspects of the professional-patient-family interpersonal relationship, and patients should, only after having been clearly clarified, decide on the treatment and conduct that they deem most appropriate to their case^(37)^.

The dilemma of not being prepared, through specialization and previous experience, and not feeling confident in providing care in a specific sector, as occurred with some frequency during the COVID-19 pandemic, was also highlighted among the mapped dilemmas. The case in question concerned professionals who worked exclusively in rescue ambulances in Texas (USA) and were transferred without proper training to a hospital Intensive Care Unit^(24,25,32,33,37)^. Certainly, the COVID-19 pandemic forced a high number of professionals to leave specialized sectors, such as Intensive Care and Emergency Units, which led to relocations; however, it is important to emphasize that it is up to each professional to identify their skills and competencies to perform their functions, in a safe way for themselves and patients, and it is up to legislators to define legal provisions that assure professionals and guarantee the appropriate quality and safety of care practices, even in the face of emergencies and disasters.

It is also important to emphasize that this reservation does not exempt professionals from later seeking knowledge from scientific evidence so that such an occurrence of refusal does not happen again, and it is up to them to formalize the situation to their immediate supervisor, in order to communicate about the need for specific training so that they can positively carry out the activities requested of them in the event of a new relocation^(38)^.

Other dilemmas mapped were: installation of a permanent airway in case of emergencies and disasters, whether the decision to perform such a procedure would be interfered with if a medical professional was at the scene; who is competent to determine the interruption of cardiopulmonary resuscitation; whether or not to allow family members to accompany emergency care/transport to a minor victim (child); and the condition of always being ready to respond to an emergency/disaster situation, even on a day off, aware of the civil and criminal responsibilities that involve this act of solidarity^(21, 28, 30)^.

In short, such dilemmas have the effect of demonstrating that: 1) nursing in emergencies and disasters is one of the most challenging and complex areas from a technical and legal point of view, given that, amid chaotic and sometimes unpredictable scenarios, nurses are responsible for providing critical and life-sustaining care; 2) nursing practice in emergencies and disasters tends to be permeated by ethical-legal dilemmas that can affect the quality of care provided and, in some cases, put patient safety at risk; 3) it is essential that these dilemmas are (re)recognized and observed to ensure that nursing practice in such situations is carried out with high ethical and legal standards.

Despite the limitation of this study of not being able to retrieve nine records and not obtaining a response from the authors of another 14, it is noted that those that were not answered were published more than 10 years ago and that, by reading the titles and abstracts, no relevant framework was observed in relation to the objective of the research.

## CONCLUSION

Mapping the ethical-legal dilemmas faced by nurses, in the context of emergencies and disasters, made it possible to identify not only the need to develop more studies on the topic, but also to structure a legal system capable of responding satisfactorily to the numerous challenges imposed on these professionals in emergencies and disasters. Such events, due to their complexity, dynamics and magnitude, have the effect of enhancing the emergence of ethical-legal dilemmas, whether due to doubts regarding legal regulations or even lack of knowledge of legal norms, which highlights the need to structure processes well of teaching nursing legislation during these professionals’ training and qualification.

The exercise of a more global look at the standards that govern health professionals, issued by the various councils and organizations of different categories, has also proven valuable so that conflicting standards regarding the same procedures can be resolved in the light of law, with the purpose of ethically and legally supporting nursing professionals’ decision.

The studies did not point out specific legislation to guide practices and support nurses through their professional practice in emergency and disaster situations, and presented as gaps certain limits of professionals’ performance within the interventions of multidisciplinary teams, specificities of practice and despite the presence of the family during assistance. It is clear that there is a need for (re)knowledge by nurses, leaders and researchers of legal limitations so that these professionals do not suffer penalties resulting from their assignment before the occurrence of new events, whether predictable or not. In other words, it is necessary to be not only prepared and attentive, but also safe by the legal system and supported by the fundamental principles that govern the profession.
